# Comparison of Temperature Distribution between TWIP and Plain Carbon Steels during Wire Drawing

**DOI:** 10.3390/ma15238696

**Published:** 2022-12-06

**Authors:** Joong-Ki Hwang

**Affiliations:** School of Mechatronics Engineering, Korea University of Technology & Education, Cheonan 31253, Republic of Korea; jkhwang@koreatech.ac.kr; Tel.: +82-041-560-1642

**Keywords:** thermal properties, wire drawing, temperature distribution, twinning-induced plasticity steel

## Abstract

The effect of the thermal properties of steels on wire drawing behavior has been investigated to understand and improve the wire drawing process. Finite element analysis and experimental tests were conducted to analyze the temperature distribution of the deformed specimens with different thermal properties. The thermal properties of twinning-induced plasticity (TWIP) steel were measured and compared with those of plain carbon steel. Based on the measurement of thermal properties, wire drawing behaviors were systematically compared with thermal conductivity of the specimen (*k*) using plain low-carbon steel with high *k* and TWIP steel with low *k*. The results revealed that the *k* of TWIP steel was approximately one third of that of low-carbon steel, and the thermal expansion coefficient of the TWIP steel was approximately 50% higher than that of low-carbon steel in the temperature range of 26–400 °C. The temperature distributions in the wire strongly depended on the *k* of the wire during wire drawing. TWIP steel exhibited higher maximum temperature, and took a longer time to attain the equilibrium temperature than low-carbon steel during wire drawing owing to the low *k*. The maximum temperature of the die increased with decreasing *k* of the wire, indicating that die wear can increase with decreasing *k* of the wire. Therefore, reducing the drawing speed is suggested for a wire with low *k*, such as high-alloyed metals, especially for TWIP steels.

## 1. Introduction

The strong demand for weight reduction in the transportation industry, particularly in steel wire, rod, and bar applications, is driving the development of stronger steels. The steels with the microstructures of pearlite, tempered martensite, and ferrite are primarily used for the wire rod products. The tensile strength of theirs has been increased with the use of the several metal strengthening techniques: modification of chemical compositions, forming processes, and heat treatments. Theoretically, controlling the chemical compositions of a metal product most effectively increases the strength of a metal product. Accordingly, research and development has been conducted for increasing the chemical compositions of the metal in order to utilize its various strengthening mechanisms. However, as the chemical composition of metals increase, the thermal properties of materials deteriorate, which may cause difficulties in the manufacturing processes, such as plastic forming, machining, and casting.

For example, Hwang et al. [1,2] suggested that twinning-induced plasticity (TWIP) steels are strong candidate for wire, rod, and bar applications, such as bolts, bearings, and springs because TWIP steels satisfy the strict property requirements of products. For example, TWIP steel has excellent combination of strength, ductility, toughness, and resistance to hydrogen-delayed fracture [3,4,5,6]. Additionally, TWIP steels can eliminate expensive heat treatment processes owing to their outstanding work hardening rate. Most researchers have attributed the outstanding mechanical properties of TWIP steels to mechanical twinning and/or dynamic strain aging that occur during plastic deformation [7,8,9].

However, TWIP steels are high-alloyed steels with Mn composition of 10–30%, and these high alloys can deteriorate the thermal properties of the material, which in turn can reduce the formability of TWIP steels, especially the wire drawability for wire, rod, and bar applications. Wire drawing is the most commonly used plastic forming process for manufacturing wire, rod, and bar products [10]. During the wire drawing process, the cross-sectional area of the wire is reduced by pulling the wire through shaped dies for attaining desired product shape and mechanical properties. The temperature of the wire, during wire drawing, is dependent on both the drawing speed and thermal properties of the metal; thus, investigating the role of drawing speed and thermal properties of the metal during wire drawing is necessary to improve the drawability and quality of wire products.

Several studies have investigated the influence of drawing speed on the drawing force, temperature rise, and mechanical properties of metals [11,12,13,14,15,16,17]. These studies have reported that an increase in the drawing speed increases the drawing force, temperature, friction between the wire and die, tensile strength, and yield strength. For instance, Lee et al. [16] reported that a temperature increase can worsen the ductility of the drawn wire and cause wire breakages during wire drawing owing to the strain aging of the wire. However, few studies have investigated the relationship between the thermal properties of a metal and wire drawing behavior although metals used as wire drawing products have various thermal properties. In addition, the thermal properties of a metal such as thermal conductivity, heat capacity, and thermal diffusivity are dependent on the temperature [18,19,20,21,22]. The author believes that the lack of such research in the past can be attributed to no attempt of making wire rod products using high-alloyed steels, such as TWIP steels, thus the thermal properties of the material are not important.

Consequently, the present study deals with the influence of the thermal properties of steels on wire drawing behavior to understand and improve the wire drawing process. Finite element analysis (FEA) and experimental tests were conducted to analyze the temperature distribution of deformed specimens with thermal properties. The thermal properties of TWIP steel were measured and compared with those of plain carbon steel. Based on the measurement of thermal properties, wire drawing behaviors were systematically compared using plain low-carbon steel with high thermal properties and TWIP steel with low thermal properties. Finally, the different wire drawing behaviors were revealed and process design concept was suggested depending on thermal properties of the wire.

## 2. Experimental Procedures and Numerical Simulation

### 2.1. Materials

A 13 mm-diameter low-carbon steel wire rod was provided by POSCO in South Korea. This wire rod was manufactured by reheating the billet at about 1200 °C, normal hot rolling at a temperature above 900 °C, and Stelmor cooling process at an average cooling rate of approximately 3 °C/s [23]. The chemical composition is listed in Table 1.

In the case of TWIP steel, a 50 kg-ingot was cast by vacuum induction melting. The chemical composition is listed in Table 1. To reduce Mn segregation in ingot, the cast ingot was homogenized at 1200 °C for 12 h in a furnace, and then directly rolled down to the of 20 mm-thick plate at a final rolling temperature of approximately 950 °C, followed by cooling in the air at temperature of 21 °C. For the wire drawing test, round rods with diameter of 13 mm were machined from the rolled plate. 

### 2.2. Wire Drawing 

**Figure 1:**
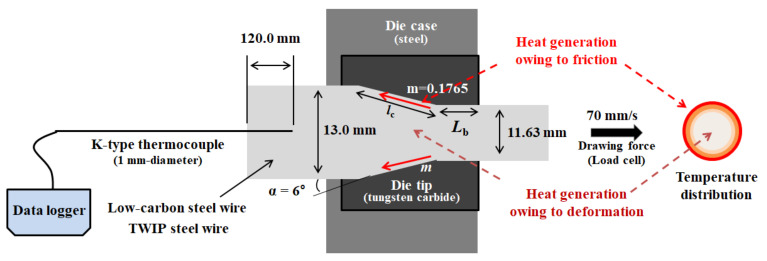
Schematic description of process conditions used in this wire drawing experiment.

The 13 mm-diameter wire rods were drawn into 11.63 mm-diameter wire with the drawing speed of 0.07 m/s using the single die-type draw bench machine at room temperature (RT, 26 °C) as shown in Figure 1. Before the drawing test, the oxidation scale on the specimen was removed by chemical pickling with 12.5% HCl, and then MoS_2_ lubricant was sprayed on the wire surface. The semi-die angle of the die was 6° and the reduction in area per pass was approximately 20%. 

The core temperature of the wire was measured using 1.0 mm-diameter sheathed K-type thermocouples. To reduce the temperature disturbances at the wire surface, a thermocouple was embedded at the tail of the specimen through a 1.0 mm-diameter hole, as shown in Figure 1 [23]. The sampling time of the data logger is 0.2 s. The drawing force was measured using a load cell in the draw bench machine.

### 2.3. Measurement of Mechanical Properties and Microstructure

Cylindrical-type tensile specimens with a gauge diameter of 5 mm and length of 25 mm were prepared along the rolling direction of the specimen. They were strained at a rate of 10^−3^ s^−1^ using an Instron machine at RT [24].

The specimens were sectioned perpendicular to the hot-rolling direction to observe their initial microstructures. Low-carbon and TWIP steels were characterized using scanning electron microscopy (SEM) and electron backscatter diffraction (EBSD), respectively. Standard mechanical polishing with diamond pastes and colloidal silica slurry polishing were applied for the samples. 

### 2.4. Measurement of Thermal Properties 

The specific heat capacity (*c*_p_), thermal diffusivity (*α*), and linear thermal expansion coefficient (*β*_L_) of the low-carbon and TWIP steels were measured in the temperature range of 26–400 °C. *c*_p_ was measured based on simultaneous thermal analysis (STA), Netzsch STA449 F5 Jupiter, Germany [25]. To prevent oxidation of the specimen, Ar flow was used during the test. The α of the steels were measured using laser flash analysis (LFA), Netzsch LFA 467 HT, Germany. The disk-shaped specimen with a diameter of 12.5 mm and thickness of 2.5 mm was used as the test specimen [26]. Ar gas was used during the test to prevent oxidation on the specimen surface. To calculate the density (*ρ*) of the two steels with temperature based on *ρ* at RT (*ρ*_0_), *β*_L_ was measured using thermomechanical analysis (TMA), TA Instruments TMA Q400, New Castle, DE, USA [27]. The heating rate was 5 °C/min and an N_2_ gas was used during the test.

### 2.5. Finite Element Analysis

**Figure 2:**
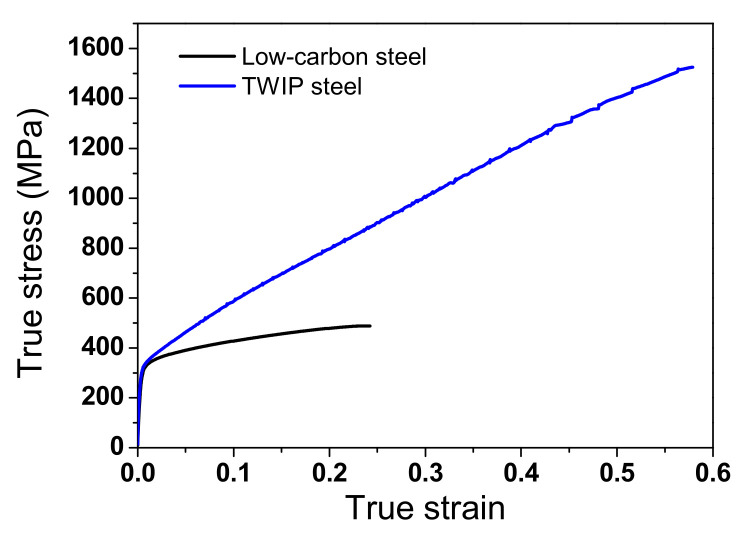
Comparison of true stress–strain curves of low-carbon and TWIP steels in tensile test.

During wire drawing, the temperature increase primarily originates from two reasons: heat generation owing to the plastic deformation of the specimen and owing to the friction between the wire and die interface as shown in Figure 1 [10]. Accordingly, the surface region of the wire exhibited a higher temperature than that of the core region. Unfortunately, measuring the surface temperature of cylindrical-type small wire rods using both non-contact radiation-type thermometers and contact conduction-type thermocouples is difficult [23]. Additionally, inhomogeneous plastic deformation occurs along the radial direction of the wire during wire drawing [28,29], resulting in a complex temperature distribution within the wire. Hence, FEA was applied to extensively evaluate the complex temperature distribution of the wire during wire drawing because it provides direct information on the complex temperature and deformation distribution in a deformed specimen. Commercial software, DEFORM (version 11.0), was applied to simulate the wire drawing process with an axi-symmetric module. Figure 2 shows the tensile true stress–strain curves of the hot-rolled low-carbon and TWIP steels. The input flow curves for FEA were acquired using these tensile stress–strain curves. The wire was considered to be isotropic material. Therefore, the constitutive behavior of the wire can be described using strain hardening coefficient (*K*) and strain hardening exponent (*n*) based on Hollomon’s law as follows:

*σ* = *Kε*^n^ (1)

Table 2 summarizes the *n* and *K* values of the two steels. These values were obtained by fitting the tensile curves of the hot-rolled steels (Figure 2). The die was taken as a rigid body, indicating that it did not deform during the entire process. 

The shear friction factor was selected as 0.1765 based on a previous investigation [30]. During plastic deformation, the temperature rise was numerically calculated using the following equation [31,32]:

(2) $$\Delta T_{r}=\frac{\Delta u}{\rho c_{p}}=\frac{\xi }{\rho c_{p}}\int_{\varepsilon_{1}}^{\varepsilon_{2}}\sigma d\varepsilon$$

where ΔT_r_, *u*, and *ξ* are the temperature increase, generated heat energy, and fraction factor between the mechanical work and heat energy, respectively. *ξ* is assumed to be 0.9 because small mechanical work was stored within the deformed specimen as elastic energy [31,32]. 

The other process conditions were identical to those used in the experiment, as shown in Figure 1. In other words, initial wire diameter, drawing speed, reduction in area per pass, and semi-die angle were 13 mm, 0.07 m/s 20%, and 6°, respectively. The thermal properties of the two steels were measured with respect to temperature, and these values were used as input parameters for the FEA. Meanwhile, the thermal conductivity (*k*) of the die with a tungsten carbide was considered to be constant at 70 W/m °C [33]. To shorten the computation time, the half of full geometry was simulated owing to the symmetrical condition of wire drawing process. The number of element in the workpiece was 10,000 and that in the die was 2000. 

## 3. Results and Discussion

### 3.1. Thermal Properties

**Figure 3:**
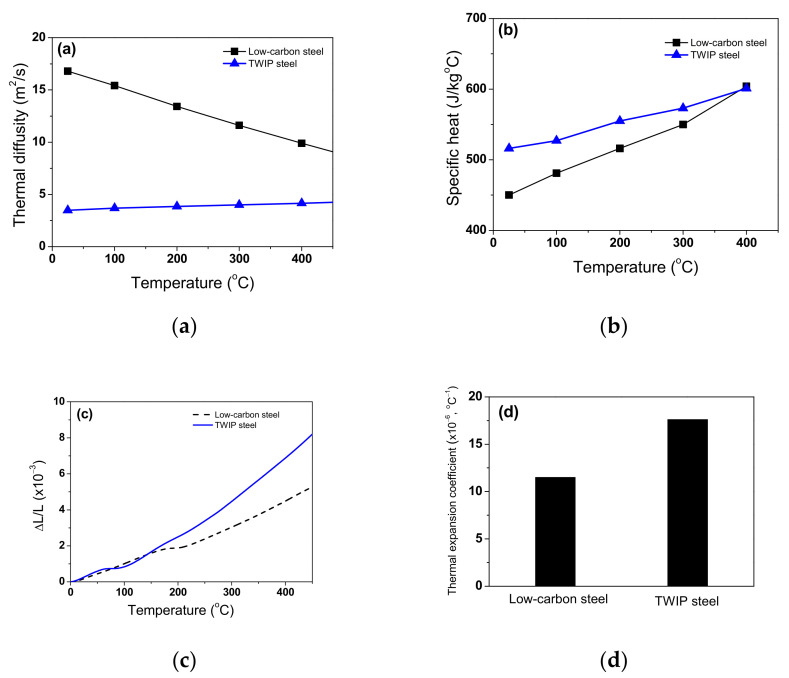
Variations in measured (**a**) thermal diffusivity, (**b**) specific heat capacity, and (**c**) expansion ratio of length as a function of temperature. (**d**) Calculated average thermal expansion coefficient of two steels based on expansion ratio of length.

The *ρ*, *c*_p_, *α*, *k*, and *β*_L_ values of a metal are dependent on temperature. Figure 3 compares the measured *α*, *c*_p_, and *β*_L_ of the low-carbon and TWIP steels as a function of temperature. *β*_L_ was calculated using the following equation [34]:

(3) $$\beta_{L}\left(T\right)=\frac{∆L}{L_{0}}\frac{1}{∆T}$$

where *L* is the length of the specimen and subscript o means the value at the reference point, i.e., at RT. Interestingly, the *β*_L_ of the TWIP steel was approximately 50% higher than that of low-carbon steel in the temperature range of 26–400 °C. *α* of low-carbon steel decreased with temperature, whereas *α* of TWIP increased with temperature within this temperature range. The *c*_p_ of both steels increased with temperature, which is consistent with the previous results with plain carbon steels [35,36]. The volume of a metal (*V*) can be calculated using the following equation:

(4) $$V={\left(L_{0}+∆L\right)}^{3}=L_{0}{}^{3}{\left(1+\frac{∆L}{L_{0}}\right)}^{3}$$

Assuming the small Δ*L/L*_0_, Equation (4) can be approximated as follows from Taylor series expansion:

(5) $$V=V_{0}\left(1+3\frac{∆L}{L_{0}}\right)$$

**Figure 4:**
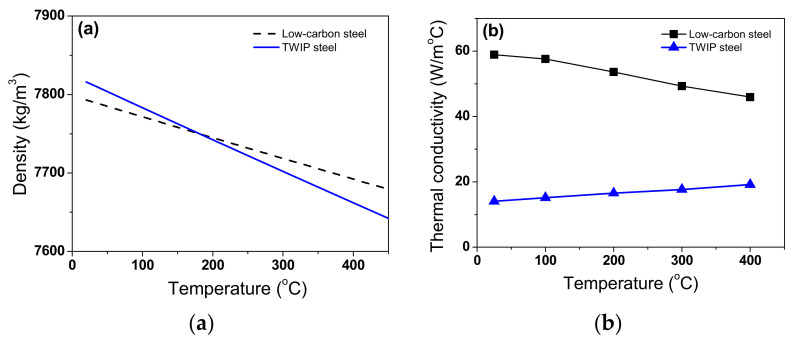
Variations in calculated (**a**) density and (**b**) thermal conductivity with temperature of two steels.

By combining Equations (3) and (5), *ρ* was calculated based on the measured *ρ*_0_ and *β*_L_ as follows:

(6) $$\rho =\frac{m}{V_{0}\left(1+3\frac{∆L}{L_{0}}\right)}=\rho_{0}\frac{1}{1+3\beta_{L}∆T}$$

where *m* is mass. As expected, *ρ* decreased with temperature, as shown in Figure 4a, which is consistent with the previous results [19,37]. Meanwhile, *ρ* of TWIP steel was more sensitive to temperature compared with low-carbon steel owing to the high *β*_L_ of TWIP steel. 

*k* was calculated based on the measured *c*_p_, *α*, and calculated *ρ* values using the following equation [38]:

(7) $$k\left(T\right)=\alpha \left(T\right)\rho \left(T\right)c_{p}\left(T\right)$$

**Figure 5:**
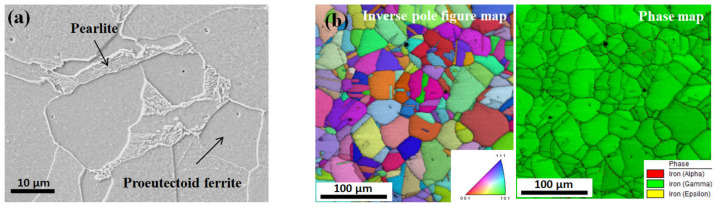
Microstructure of the hot-rolled (**a**) low-carbon and (**b**) TWIP steels used in this study.

Interestingly, the difference in *k* between the two steels was quite high as shown in Figure 4b. The *k* of low-carbon steel decreased with temperature, which is consistent with the previous results of plain carbon steels with body-centered cubic (BCC) structure [18,21]. By contrast, the *k* of TWIP steel increased with temperature, which is consistent with the results of stainless steels with face-centered cubic (FCC) structure [22,39]. The *k* of TWIP steel was approximately one third that of low-carbon steel. The differences in *k* and *β*_L_ between the two steels were related to their chemical compositions, crystal structures, and microstructures. The crystal structures of low-carbon and TWIP steels are BCC and FCC structures [7] in this temperature range, respectively. Compared with low-carbon steel, high-alloy content of TWIP steel (Table 1) impaired the thermal properties, particularly the *k* value. In addition, the different microstructures of the TWIP and low-carbon steels affect their thermal properties. Low-carbon steel is composed of a ferritic microstructure with a small amount of pearlite, as shown in Figure 5a. In the TWIP steel, a recrystallized grain with an average grain size of 41 μm was observed based on the EBSD inverse pole figure map, and only the FCC structure appeared from the phase map (Figure 5b). The different *c*_p_, *ρ*, and *k* of the two steels are summarized as a function of temperature in Table 3. These thermal properties were used as the input parameters to simulate the wire drawing process. 

### 3.2. Model Validation 

**Figure 6:**
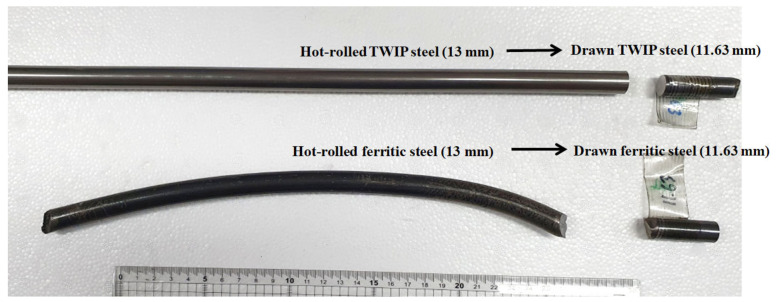
Photograph of the hot-rolled and drawn low-carbon and TWIP steel wires.

Before analyzing the results of the FEA of the drawn wire with the thermal properties, the accuracy of the FEA model was validated by comparing the measured and simulated drawing forces and core temperatures of the two steels. Figure 6 shows the photograph of the hot-rolled and drawn low-carbon and TWIP steel wires.

**Figure 7:**
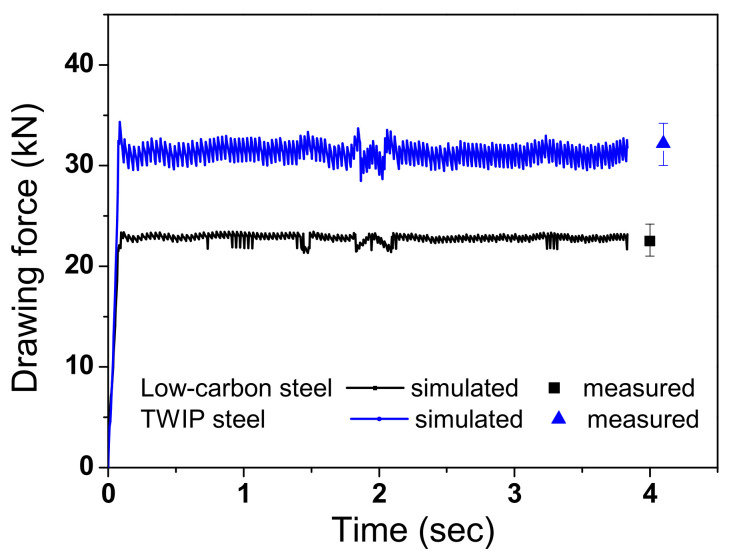
Comparison of the equilibrium drawing forces between experiment and FEA in low-carbon and TWIP steels.

Figure 7 shows a comparison of the numerically simulated and experimentally measured drawing forces of the two steels. Small fluctuations were observed with time in both the measured and calculated drawing forces. The numerically simulated drawing forces were in good agreement with the measured equilibrium values. The drawing force of TWIP steel was higher than that of the low-carbon steel. 

**Figure 8:**
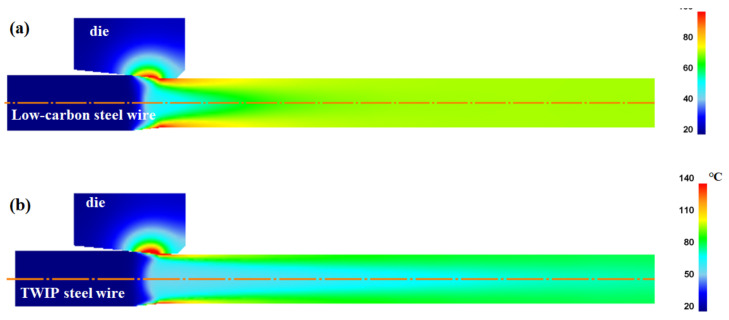

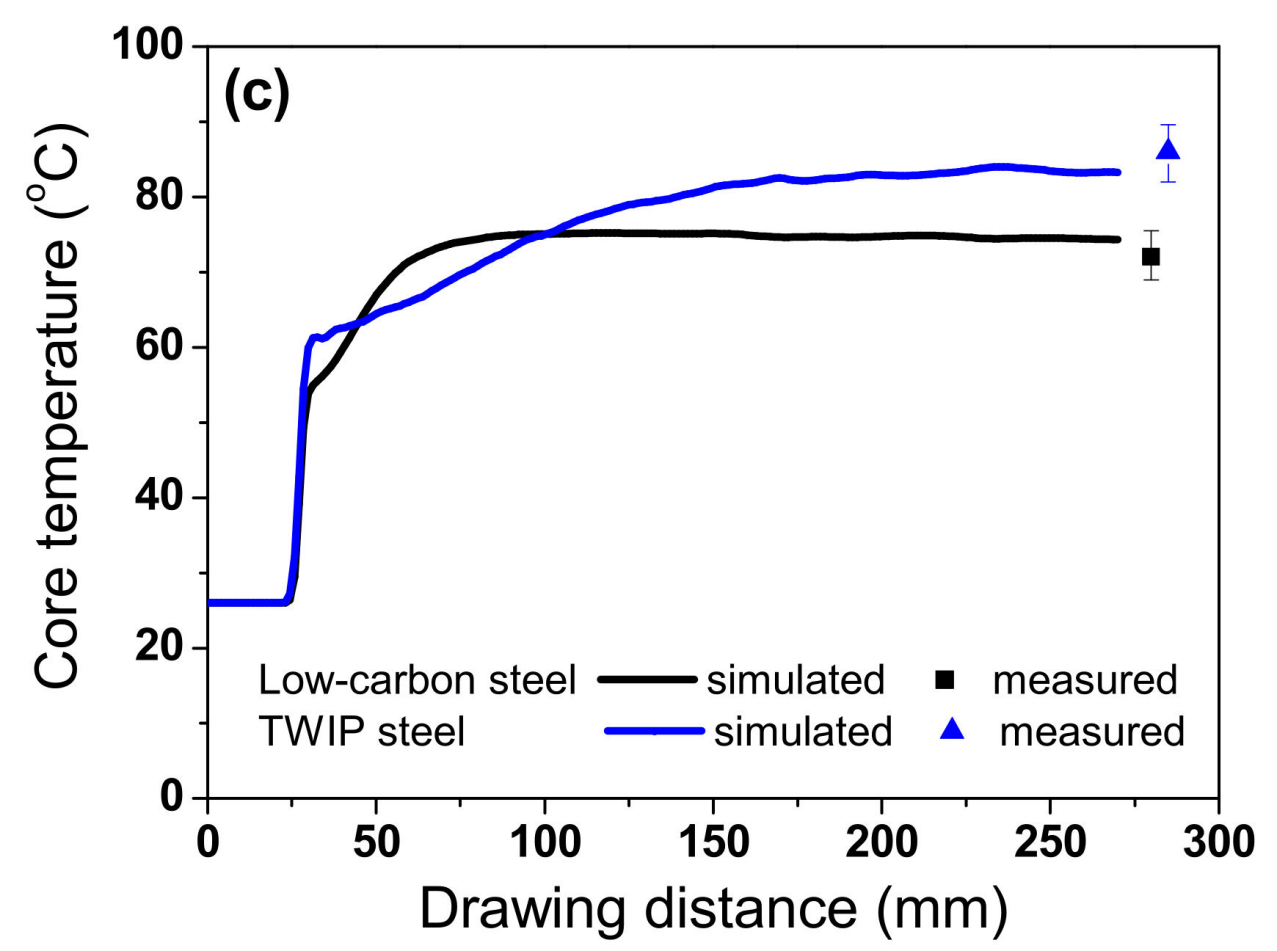
Comparison of temperature contour of (**a**) low-carbon steel and (**b**) TWIP steel, and (**c**) equilibrium core temperatures between experiment and FEA of two steels.

Figure 8 also compares the calculated equilibrium temperature (T_eq_) with the FEA and measured T_eq_ with the thermocouple. The calculated T_eq_ of the wire was in good agreement with the measured T_eq_. The temperature rise of the two steels exhibited a pattern similar to that of the drawing force. The T_eq_ of TWIP steel was higher than that of low-carbon steel. The detailed analysis of temperature will be discussed in result section. Based on the validation of the drawing force and core temperature, the results of the current FEA were concluded to be acceptable and reliable for further analysis.

### 3.3. Wire Drawing Behaviors with Thermal Properties of a Wire

**Figure 9:**
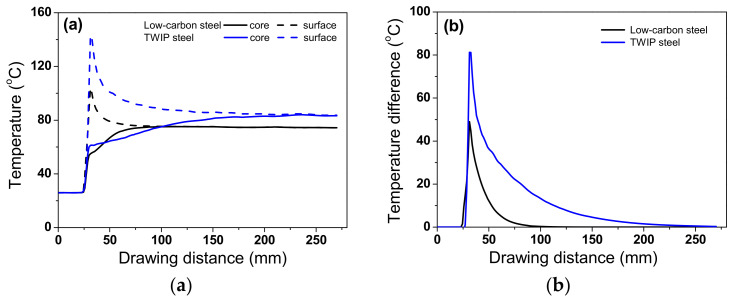
Comparison of numerical simulation results of (**a**) temperature profiles along the drawing direction at core and surface regions and (**b**) temperature difference between core and surface regions of low-carbon and TWIP steels.

Figure 9a compares the numerical simulation results of the temperature profiles along the drawing direction at the core and surface regions of the two steels, based on the temperature contour in Figure 8. The temperature gradient existed along the radial direction of the wire because the frictional heating at the surface region resulted in a steep increase in the temperature at the surface region of the wire [40]. In both steels, the core temperature gradually increased, while the surface temperature rapidly increased and then gradually decreased because the heat by frictional stress was transferred to the specimen interior by the conduction heat transfer mechanism until the specimen temperatures attained equilibrium. Additionally, ambient air cooled the specimen surface by the convection and radiation heat transfer mechanisms although the effect was relatively insignificant. Meanwhile, T_eq_ of TWIP steel was higher than that of low-carbon steel. Figure 9b compares the temperature difference between the core and surface regions of the low-carbon and TWIP steels. The temperature difference (ΔT_D_) was defined as follows:

(8) $$∆T_{D}=T_{s}-T_{c}$$

where T_s_ and T_c_ indicate temperatures in the surface and core regions, respectively. ΔT_D_ of TWIP steel was higher than that of low-carbon steel. Additionally, TWIP steel required a longer time to attain T_eq_ compared with low-carbon steel. These two phenomena can be related to the difference in the thermal properties of the two steels. The relatively high *k* of the low-carbon steel rapidly transferred the heat generated by the frictional stress into the wire interior and ambient environment. By contrast, the speed of heat conduction within the TWIP steel wire was relatively low owing to its low *k*.

**Figure 10:**
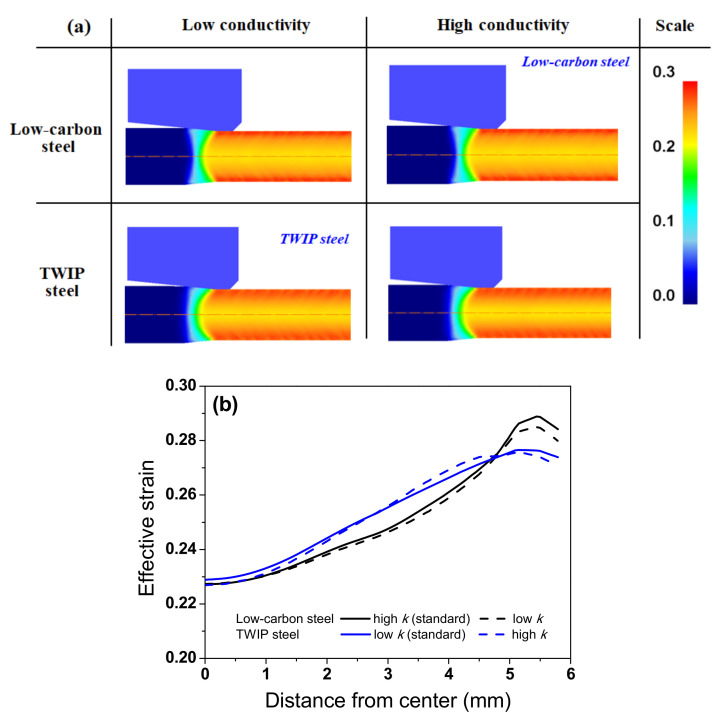
Comparison of effective strain (**a**) contours and (**b**) profiles along the radial direction of low-carbon and TWIP steels with different thermal conductivity.

Figure 10 compares the effective strain of the two steels with different k. For a better comparison, the effective strain distributions of TWIP and low-carbon steels with low and high *k* were compared. In this study, a high *k* means the *k* of the low-carbon steel (*k*_high_), and a low *k* means the *k* of TWIP steel (*k*_low_). In other words, additional numerical simulations were performed for TWIP and low-carbon steels with the different *k* values. All wires had the maximum and minimum effective strains near the surface and at the core, respectively, which is consistent with the previous results [41,42]. During wire drawing, the temperature increase by the plastic deformation was higher in the surface region than that in the core region owing to the higher plastic deformation based on Equation (2). Meanwhile, TWIP steel exhibited a more uniform strain distribution along the radial direction of the wire regardless of *k* values compared with low-carbon steel, which is highly related to the high *n* value of TWIP steel [43,44,45]. Based on this result, the higher ΔT_D_ in TWIP steel compared with low-carbon steel (Figure 9) was not related to the strain distribution. In addition, the influence of *k* on the effective strain distribution of the wire can be concluded to be insignificant based Figure 10b. 

**Figure 11:**
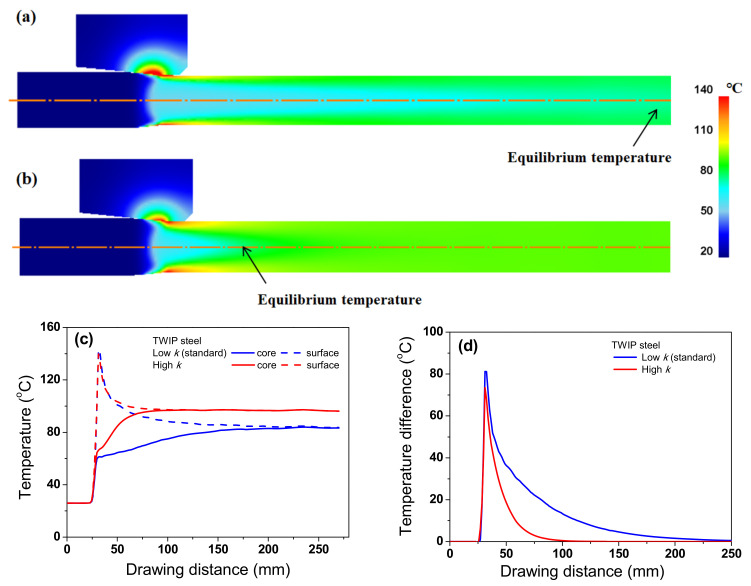
Comparison of temperature contour of TWIP steel with (**a**) standard and (**b**) high thermal conductivities. Comparison of (**c**) temperature profiles along the drawing direction at core and surface regions and (**d**) temperature difference between core and surface regions of TWIP steel.

**Figure 12:**
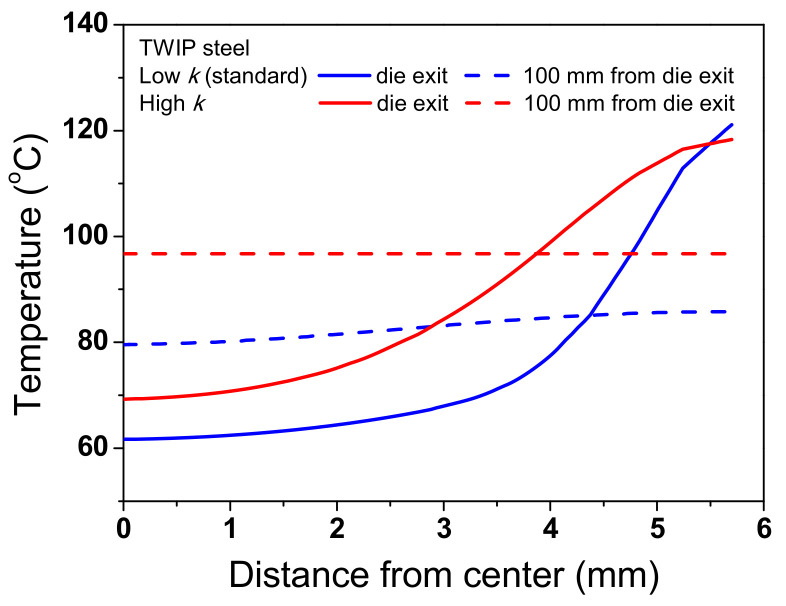
Comparison of temperature profiles along the radial direction of TWIP steel at die exit and 100 mm from die exit with different thermal conductivities.

**Figure 13:**
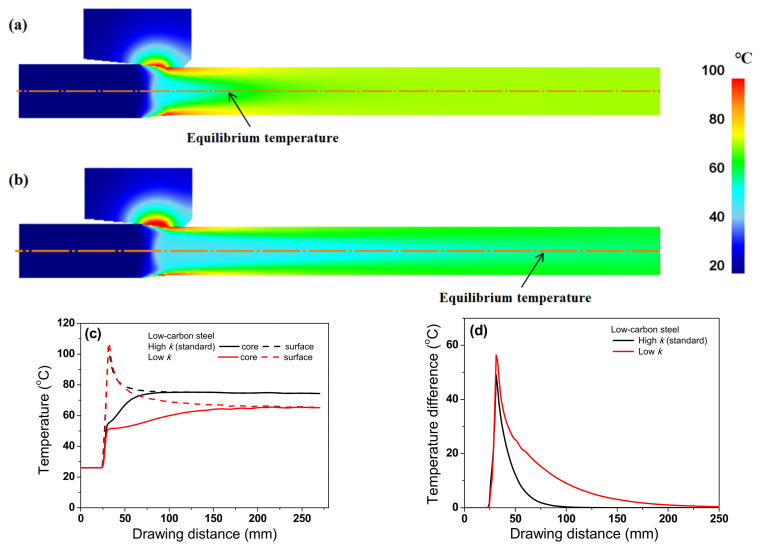
Comparison of temperature contour of low-carbon steel with (**a**) standard and (**b**) low thermal conductivities. Comparison of (**c**) temperature profiles along the drawing direction at core and surface regions and (**d**) temperature difference between core and surface regions of low-carbon steel.

**Figure 14:**
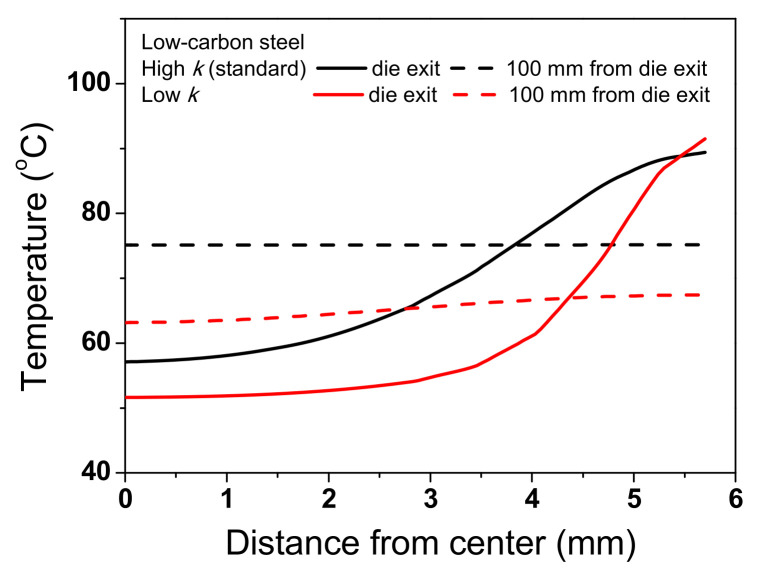
Comparison of temperature profiles along the radial direction of low-carbon steel at die exit and 100 mm from die exit with different thermal conductivities.

To thoroughly investigate the effect of *k* on the temperature distribution of the wire, the temperature distributions of the TWIP steel with *k*_low_ and *k*_high_ were compared, as shown in Figure 11. The temperature distributions were different with respect to the *k* of the specimen during wire drawing. The maximum temperature was higher, and the necessary time for temperature equilibrium in the specimen increased as *k* of the specimen decreased. Accordingly, ΔT_D_ increased with decreasing *k*, which was strongly confirmed by analyzing the temperature profile along the radial direction of the wire during wire drawing as shown in Figure 12. The temperature distribution of the wire with k_high_ was more uniform compared with the wire with *k*_low_. It was confirmed that this phenomenon also appears in low-carbon steel as shown in Figure 13 and Figure 14 although the absolute values of temperature differed with material depending on other material properties, such as *K* and *n* values. Finally, it can be concluded that the temperature gradient along the radial direction of the specimen is strongly dependent on the thermal properties of the specimen during wire drawing. 

### 3.4. Die Temperature with Thermal Properties of Wire

**Figure 15:**
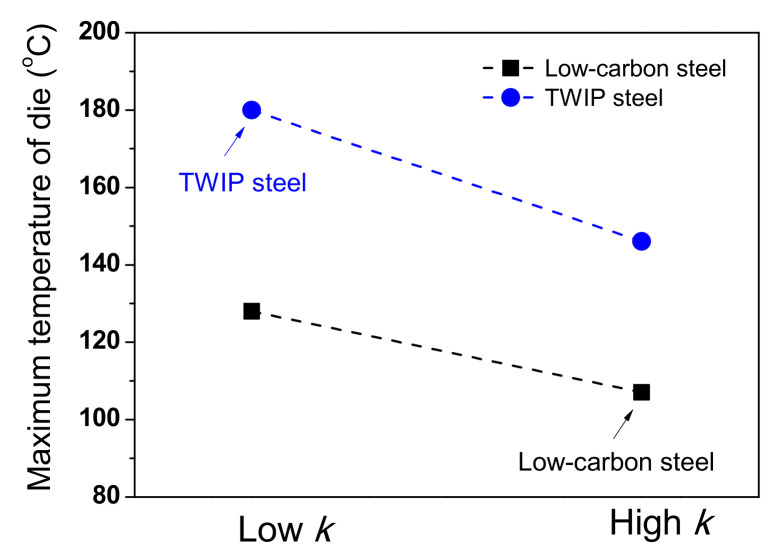
Comparison of maximum temperature of die for low-carbon and TWIP steels with different thermal conductivities based on Figure 11 and Figure 13.

**Figure 16:**
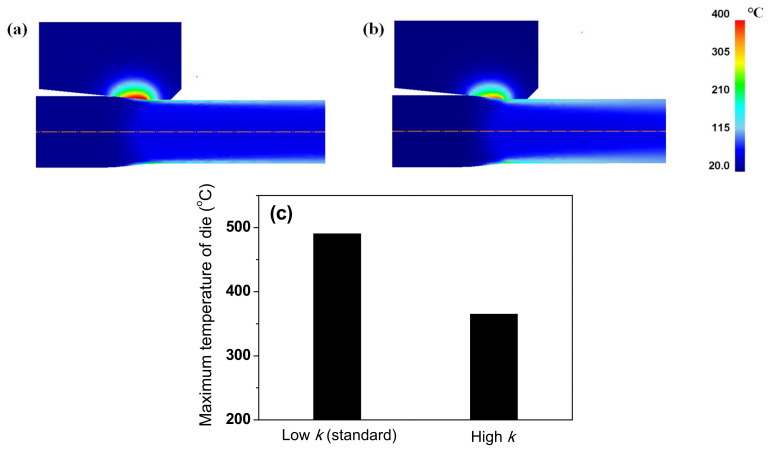
Comparison of temperature contour of TWIP steel with (**a**) low and (**b**) high thermal conductivities at drawing speed of 0.7 m/s. (**c**) Comparison of maximum temperature of die between two drawing conditions.

During wire drawing, die wear is strongly related to the temperature distribution of the die [46]. Therefore, analyzing the die temperature with the *k* value of a metal is necessary. Figure 15 summarizes the maximum temperature of the die for low-carbon and TWIP steels with different *k* values. The maximum temperature of the die increased with decreasing *k* of the wire, indicating that die wear can increase with decreasing the *k* of the wire. Figure 16 shows the comparison of temperature contour of TWIP steel with *k*_low_ and *k*_high_ at the drawing speed of 0.7 mm/s to simulate the working conditions in a real plant. Both the wire and die temperatures increased with decreasing *k* of the wire, leading to an increase in die wear. The die temperature increased with decreasing *k* of the wire because the heat from the frictional heating effect between the wire and die was easily transferred to the die rather than the wire owing to the low *k* of the wire. Therefore, it is necessary to reduce the drawing speed as the *k* of the wire decreases. For example, the drawing speed should be decreased in TWIP steels compared with plain carbon steels. Based on the experiences of the author, the die wear was much higher in TWIP steel than in plain carbon steels during wire drawing. It should be noted that the die temperature in real plants was less than 300 °C owing to the cooling process in both the die and wire. Meanwhile, further study regarding the effect of thermal properties of a die on the wire drawing behaviors is necessary. 

## 4. Conclusions

Based on a comparative study of the influence of the thermal properties of metals on the wire drawing behavior, the following conclusions were derived:

1. The thermal conductivity (*k*) of TWIP steel was approximately one third of that of plain low-carbon steel, and the thermal expansion coefficient of the TWIP steel was approximately 50% higher than that of low-carbon steel in the temperature range of 26–400 °C.
2. The temperature distributions of the wire strongly depended on the *k* of the wire during wire drawing. The maximum temperature of TWIP was higher, and the TWIP steel took a longer time to attain the equilibrium temperature within the wire compared with the low-carbon steel during wire drawing owing to the low *k*.
3. The maximum temperature of the die increased as the *k* of the wire decreased, indicating that die wear can increase with decreasing the *k* of the wire. Therefore, it is suggested to reduce the drawing speed as the *k* of the wire decreases, especially in high-alloyed metals and TWIP steels. Meanwhile, further study regarding the effect of thermal properties of a die on the wire drawing behaviors is necessary.

## Figures and Tables

**Table 1:** Chemical compositions of the low-carbon and TWIP steels used in this study.

Steels		Compositions (wt.%)			
	C	Mn	Si	Cu	Fe
Low-carbon	0.10	0.40	0.10	-	Balance
TWIP	0.72	17.07	-	2.90	Balance

**Table 2:** Flow stress of wire and die used in this simulation.

Material	Flow Stress (MPa)
Low-carbon steel	*σ* = 628*ε*^0.16^
TWIP steel	*σ* = 1984*ε*^0.53^
Tungsten carbide die	Rigid body

**Table 3:** Thermal properties of the low-carbon and TWIP steels with temperature.

Steels	Specific Heat [*c*_p_] (J/kg °C)	Density [*ρ*] (kg/m^3^)	Thermal Conductivity [*k*] (W/m °C)
Low-carbon steel	438.8 + 0.40*T* (°C)	7798.0 − 0.26*T* (°C)	60.5 − 0.036*T* (°C)
TWIP steel	507.6 + 0.23*T* (°C)	7823.3 − 0.40*T* (°C)	13.7 + 0.013*T* (°C)

## Data Availability

Not applicable.

## References

[1] Hwang J.K., Yi I.C., Son I.H., Yoo J.Y., Kim B., Zargaran A., Kim N.J. (2015). Microstructural evolution and deformation behavior of twinning-induced plasticity (TWIP) steel during wire drawing. Mater. Sci. Eng. A.

[2] Kim S.J., Lee T., Hwang J.K. (2020). High-strength bolt manufactured by an extrusion-based forming process using twinning-induced plasticity steel. J. Manuf. Process..

[3] Bouaziz O., Allain S., Scott C.P., Cugy P., Barbier D. (2011). High manganese austenitic twinning induced plasticity steels: A review of the microstructure properties relationships. Curr. Opin. Solid State Mater. Sci..

[4] Grassel O., Kruger L., Frommeyer G., Meyer L.W. (2000). High strength Fe-Mn-(Al, Si) TRIP/TWIP steels development-properties-application. Int. J. Plast..

[5] Chun Y.S., Lee J., Bae C.M., Prak K.T., Lee C.S. (2012). Caliber-rolled TWIP steel for high-strength wire rods with enhanced hydrogen-delayed fracture resistance. Scr. Mater..

[6] So K.H., Kim J.S., Chun Y.S., Park K.T., Lee Y.K., Lee C.S. (2009). Hydrogen delayed fracture properties and internal hydrogen behavior of a Fe-18Mn-1.5Al-0.6C TWIP steel. ISIJ Int..

[7] De Cooman B.C., Estrin Y., Kim S.K. (2018). Twinning-induced plasticity (TWIP) steels. Acta Mater..

[8] Lee Y.K. (2012). Microstructural evolution during plastic deformation of twinning-induced plasticity steels. Scr. Mater..

[9] Luo Z.C., Huang M.X. (2020). The role of interstitial carbon atoms on the strain-hardening rate of twinning-induced plasticity steels. Scr. Mater..

[10] Wright R.N. (2011). Wire Technology: Process Engineering and Metallurgy.

[11] Vega G., Haddi A., Imad A. (2009). Temperature effects on wire-drawing process: Experimental investigation. Int. J. Mater. Form..

[12] Haddi A., Imad A., Vega G. (2011). Analysis of temperature and speed effects on the drawing stress for improving the wire drawing process. Mater. Des..

[13] El-Domiaty A., Kassab S.Z. (1998). Temperature rise in wire-drawing. J. Mater. Process. Technol..

[14] Hwang J.K. (2020). Effect of drawing speed on microstructure distribution and drawability in twinning-induced plasticity steel during wire drawing. J. Iron Steel Res. Int..

[15] Suliga M., Kruzel R., Garstka T., Gazdowicz J. (2015). The influence of drawing speed on structure changes in high carbon steel wires. Metalurgija.

[16] Lee S.K., Ko D.C., Kim B.M. (2009). Pass schedule of wire drawing process to prevent delamination for high strength steel cord wire. Mater. Des..

[17] Nemec I., Golis B., Pilarczyk J.W., Budzik R., Waszkielewicz W. (2007). Effect of high-speed drawing on properties of high-carbon steel wires. Wire J. Int..

[18] Xing Y., Wang W., Al-azzani H. (2021). Assessment of thermal properties of various types of high-strength steels at elevated temperatures. Fire Saf. J..

[19] Denis S., Sjöström S., Simon A. (1987). Coupled temperature, stress, phase transformation calculation. Metall. Mater. Trans. A.

[20] Abouelregal A.E. (2022). A comparative study of a thermoelastic problem for an infinite rigid cylinder with thermal properties using a new heat conduction model including fractional operators without non-singular kernels. Arch. Appl. Mech..

[21] Wang K.Y., Jin Y.J., Xu M.J., Chen J.S., Lu H. (2015). Estimation of heat transfer coefficient and phase transformation latent heat by modified pattern search method. Int. Commun. Heat Mass Transf..

[22] Faini F., Attanasio A., Ceretti E. (2018). Experimental and FE analysis of void closure in hot rolling of stainless steel. J. Mater. Process. Technol..

[23] Hwang J.K. (2018). The temperature distribution and underlying cooling mechanism of steel wire rod in the Stelmor type cooling process. Appl. Therm. Eng..

[24] Hwang J.K. (2019). Effects of diameter and preparation of round shaped tensile specimen on mechanical properties. Mater. Sci. Eng. A.

[25] Yu Y., Cai X., Cao Z., Jiao X., Xie W., Yu Y., Feng P. (2022). Effect of the heating rate on the thermal explosion behavior and oxidation resistance of 3D-structure porous NiAl intermetallic. Mater. Charact..

[26] Steau E., Mahendran M., Poologanathan K. (2020). Elevated temperature thermal properties of carbon steels used in cold-formed light gauge steel frame systems. J. Build. Eng..

[27] Debelak B., Lafdi K. (2007). Use of exfoliated graphite filler to enhance polymer physical properties. Carbon.

[28] Hwang J.K., Son I.H., Yoo J.Y., Zargaran A., Kim N.J. (2015). Effect of reduction of area on microstructure and mechanical properties of twinning-induced plasticity steel during wire drawing. Met. Mater. Int..

[29] Hasani G.H., Mahmudi R., Karimi-Taheri A. (2010). On the strain inhomogeneity in drawn copper wires. Int. J. Mater. Form..

[30] Moon C., Kim N. (2012). Analysis of wire-drawing process with friction and thermal conditions obtained by inverse engineering. J. Mech. Sci. Technol..

[31] Curtze S., Kuokkala V.T. (2010). Dependence of tensile deformation behavior of TWIP steels on stacking fault energy, temperature and strain rate. Acta Mater..

[32] Yang H.K., Zhang Z.J., Dong F.Y., Duan Q.Q., Zhang Z.F. (2014). Strain rate effects on tensile deformation behaviors for Fe-22Mn-0.6C-(1.5Al) twinning-induced plasticity steel. Mater. Sci. Eng. A.

[33] Felder E., Levrau C., Mantel M., Dinh N.G.T. (2012). Identification of the work of plastic deformation and the friction shear stress in wire drawing. Wear.

[34] Watanabe H., Yamada N., Okaji M. (2004). Linear thermal expansion coefficient of silicon from 293 to 1000 K. Int. J. Thermophys..

[35] Casal J.M., Porteiro J., Míguez J.L., Vázquez A. (2015). New methodology for CFD three-dimensional simulation of a walking beam type reheating furnace in steady state. Appl. Therm. Eng..

[36] García A.M., Colorado A.F., Obando J.E., Arrieta C.E., Amell A.A. (2019). Effect of the burner position on an austenitizing process in a walking-beam type reheating furnace. Appl. Therm. Eng..

[37] Huiping L., Guoqun Z., Shanting N., Chuanzhen H. (2007). FEM simulation of quenching process and experimental verification of simulation results. Mater. Sci. Eng. A.

[38] Incropera F.P., Dewitt D.P., Bergman T.L., Lavine A.S. (2006). Fundamentals of Heat and Mass Transfer.

[39] Mayrhofer M., Koller M., Seemann P., Prieler R., Hochenauer C. (2022). CFD investigation of a vertical annealing furnace for stainless steel and non-ferrous alloys strips—A comparative study on air-staged & MILD combustion. Therm. Sci. Eng. Prog..

[40] Kemp I.P., Pollard G., Bramley A.N. (1985). Temperature distributions in the high speed drawing of high strength steel wire. Int. J. Mech. Sci..

[41] Vega G., Haddi A., Imad A. (2009). Investigation of process parameters effect on the copper-wire drawing. Mater. Des..

[42] Chin R.K., Stelf P.S. (1995). A computational study of strain inhomogeneity in wire drawing. Int. J. Mach. Tools Manuf..

[43] Eom J.G., Son Y.H., Jeong S.W., Ahn S.T., Jang S.M., Yoon D.J., Joun M.S. (2014). Effect of strain hardening capability on plastic deformation behaviors of material during metal forming. Mater. Des..

[44] Kim H.S., Seo M.H., Hong S.I. (2000). On the die corner gap formation in equal channel angular pressing. Mater. Sci. Eng. A.

[45] Hwang J.K. (2022). Strain and strain rate hardening effects on the macroscopic shear bands and deformation shape of a caliber-rolled wire. J. Manuf. Process..

[46] Kim T.H., Kim B.M., Choi J.C. (1997). Prediction of die wear in the wire-drawing process. J. Mater. Process. Technol..

